# Man and His Enemies

**Published:** 1889-04-06

**Authors:** 


### The Doctor's Pulpit.

### MAN AND HIS ENEMIES.

### The "Pinafore" Period.

If a grown-up man of any considerable originality of mind were asked whether he would prefer to be a three-months' old African lion, or a twelve-months' old British infant, the chances are ten to one that he would choose to be the lion. The absolutely unchecked freedom of the young monarch of the forest must be delightful. The poor infant Briton, with his shoes and stockings, his bandages and stays, his strings and petticoats, his pins and his pinafores, his "bibs and tuckers," his baths and his brushings, his nurses and his perambulators, his aunts and grandmothers, his "powders" and puddings, and the other thousand and one fads and fancies of an artificial civilisation, must, if he can think at all, look upon his surroundings and environments as the worst possible set of circumstances in which an unkind Nature, in the unkindest of her moods, could have placed him. The writer has a little niece of two years old in Africa. Her parents attach a quite exaggerated value to most of the ways of civilisation, and take serious pains to bring up the infant in the conventional "way she should go." But Miss Two-years-old will have none of it. The free ways of the woolly-haired negro infants are altogether to her liking, and accordingly she insists upon speaking the native language, dressing as nearly as possible in the native costume, eating her food with her fingers, and slinging her dolls in a broad kerchief across her shoulders exactly as the native mothers sling their babies, and as their little female imitators of two or three years old who have no dolls, sling instead a potato or a "cob" of maize. So doing she is supremely happy; restrained within the customary European bounds and limits, she is the picture of injured despair.

A crusade is a serious business to enter upon. Yet the blessings of the whole kingdom of infancy would come upon the head of any man who should formulate and get carried a paternal Reform Bill for the deliverance of little boys and girls in the "pinafore period" from certain conventional slaveries which make their little lives "not worth living." The town child—what a demure, hopeless, unnatural, ridiculous, thirty years old little prig it is! If everything injurious is to be looked upon as a conscious or unconscious enemy, the civilised infant must be regarded as surrounded with enemies in every circumstance of life. Let us go into the nursery. Nurse is putting a bandage more or less tightly round the child's abdomen. What for? Does the child need it either for warmth or support? Most certainly not. It is neither more nor less than an injury and a nuisance. The bandage put on and fixed, what comes next? A pair of stays! Stays! What is to be stayed? Has Nature not provided the infant with ribs? Do not these ribs need to expand from twenty to thirty times a minute in respiration? What does the nurse mean, then, by putting on a pair of inelastic stays which compel the child to wear out its nerve energy in expanding them twelve hundred times an hour, and nearly twenty-nine thousand times a day? Stays and bandages for young children not only cramp their liberty, and so diminish their pleasure, but they are mischievous to health in a very decided degree. Of course, our superior mothers, whose religion is custom and whose priest is the milliner, will attach no importance to what the doctor may say; or at least they will not think of acting contrary to their religion of custom, or of disobeying the milliner, their priest. But they ought at any rate to understand that in thus acting they may be properly described by a word of one syllable, which it is very easy to spell. Children's dresses should be as loose and elastic as possible, so that the little "animals" (the word is used advisedly) may roll and tumble about at their pleasure with absolute freedom from all restraint.

What malignant demon of civilisation invented children's evening parties? There is no reason why boys and girls of all ages should not meet and play and eat together now and again at suitable times. But many parents will have gatherings of mere infants, from two years old and upwards. They will stuff them with tea and cake at five o'clock; then try to amuse them with magic-lanterns, dances, and knowbody knows what, until half-past eight or nine; and after all that they will finish off with supper. There are children who are allowed to attend parties of this kind every week or two during the whole winter. That the parents of such children are worse than lunatics goes without saying. If labelling them "mad" would do any good, there would be no difficulty in finding doctors to certify their condition. That is not where the difficulty is. The real misery and grief of it lies here, that nothing can be done to restrain such parents. The children are theirs, the State cannot interfere, and if they choose to ruin them physiologically, and mentally, too, that is solely their own look out. Someone has said "we have no children now." No; and what is more serious than that, "no children" means no men and women, but only baby prigs, grown-up conventionalities, and aged imbeciles. That is the order of it. If mere infants must meet together, it should be in small numbers, in large rooms, and in the daytime. Let them have such romps and games as they understand and take delight in, and such food as will give them pleasure, but none of the pain and danger of dyspepsia; and let them not fail to be at home and in bed punctually at the hour they are accustomed to.

Early school is another device of the enemy for checking the natural development of child life and for injuring health. The exercise and training of the mind, even of a young child, is not injurious, but, on the contrary, beneficial, provided always that it is of the right kind. Children between the ages of three and seven should be taught after the kindergarten method. It is more advantageous for them to go to school than to have home lessons only. The meeting together of various minds and characters, even though they be but child minds, is stimulating and wholesome. It is a great pity, however, that so many of the kindergartens to which young children of the more prosperous classes are sent should have such miserably inadequate school buildings. In the average kindergarten the lower rooms of a private house of moderate dimensions are generally converted into one or more classrooms, and into those stuffy apartments twenty, or from that to forty, young children, and three or four governesses, are crammed for several hours a day. Windows are of necessity frequently opened, and, underneath a falling cascade of cold wintry or spring air, the little creatures stand gasping for breath and contracting colds in the head, pleurisies, pneumonias, and other dangerous diseases. There is often no attempt of any kind made to secure a uniform supply of fresh air or an equable temperature. These are defects of great seriousness, and demand the most thorough and searching attention on the part of all who have children, or who take thought for their well-being. But given large, airy, and properly-warmed apartments, with patient and intelligent teachers, the kindergarten system is admirably adapted both for training the mind and developing the body. To the children themselves it is what education always should be—at any rate in childhood—a combination of wholesome excitement with very gentle discipline.

The "pinafore" period ought to be to every child a period of perfect health and of unalloyed happiness. With care on the part of parents and nurses, all disorders of the stomach and bowels and of the respiratory organs may generally be avoided. Even the contagious and infectious fevers may often be kept out of the home for years with proper watchfulnesss and good sanitary management. It must be understood that the cold air is an enemy or a friend according as it is managed; that nurseries and bedrooms may be wholesome or poisonous; that food may be nourishing or injurious; that exercise may strengthen or weaken; that school may do good or harm; that uncles and aunts and grandmothers may be friends or enemies; that, in short, good sense, duty, and watchfulness are imperatively demanded from parents and servants alike, if the health and happiness of the little ones—and with it the joy and the delight of the home—are to be preserved. Those who make the lives of children happy perform no mean function in the providential order of the world.

